# Supplementary material for: Food Desires and Hedonic Discrimination in Virtual Reality Varying in Product–Context Appropriateness among Older Consumers
Source: Foods. 2022 Oct 15;11(20):3228. doi: 10.3390/foods11203228 (PMC9602428; doi:10.3390/foods11203228)
Supplement: Supplementary file 1 [file foods-11-03228-s001.zip › foods-1890630-supplementary.pdf]

Supplementary Table S1. Background information of the participants (n = 70, depicted as mean ± SD or n).

	Participants
Gender (female/male)	44/26
Age	69.9 ± 4.7
Height (cm)	169.7 ± 8.2
Weight (kg)	74.8 ± 13.5
BMI (kg/m^2)	25.9 ± 4.8
Self-rated health status [1]	4.3 ± 0.6
Exercise frequency [2]	4.0 ± 1.0
Living situation	
Living alone	32
Living together	38
Education level [3]	
Low	6
Medium	20
High	44
Frequency of eating rye bread [2]	4.9 ± 0.5
Perceived healthiness of rye bread [4]	4.7 ± 0.5
Perceived healthiness of protein-enriched rye bread [4]	3.8 ± 0.8
Willingness to consume protein-enriched rye bread [4]	3.6 ± 1.1
Familiarity with VR [5]	-0.2 ± 1.3

Notes: [1] the self-rated health status was measured on a 5-point scale, of which 1 = very bad, 3 = neither good nor bad, 5 = very good. [2] The frequency of exercise and eating rye bread was measured on a 5-point scale, of which 1 = never, 2 = seldom, 3 = a few times a month, 4 = a few times a week, 5 = daily. [3] Educational level: low = primary, middle, and high school; middle = vocational school or technical college; high = university education. [4] A 5-point Likert scale of agreement was used for the three measurements: 1 = extremely disagree; 3 = neither agree nor disagree; 5 = extremely agree. [5] The familiarity with the VR headset was assessed on a 7-point scale ranging from "-3 = had never heard about it" to "+3 = being extremely familiar".
